# A Differential Resonant Accelerometer with Low Cross-Interference and Temperature Drift

**DOI:** 10.3390/s17010178

**Published:** 2017-01-18

**Authors:** Bo Li, Yulong Zhao, Cun Li, Rongjun Cheng, Dengqiang Sun, Songli Wang

**Affiliations:** 1 State Key Laboratory for Manufacturing System Engineering, Xi’an Jiaotong University, Xi’an 710049, China; li.bo.123.666@stu.xjtu.edu.cn (B.L.); licun9@stu.xjtu.edu.cn (C.L.); crj_2007@stu.xjtu.edu.cn (R.C.); sundengqiang@stu.xjtu.edu.cn (D.S.); 2 Aviation Key Laboratory of Science and Technology on Inertia, Flight Automatic Control Research Institute, Xi’an 710065, China; 618gdb104@facri.com

**Keywords:** accelerometer, quartz resonator, low temperature drift, low cross-interference

## Abstract

Presented in this paper is a high-performance resonant accelerometer with low cross-interference, low temperature drift and digital output. The sensor consists of two quartz double-ended tuning forks (DETFs) and a silicon substrate. A new differential silicon substrate is proposed to reduce the temperature drift and cross-interference from the undesirable direction significantly. The natural frequency of the quartz DETF is theoretically calculated, and then the axial stress on the vibration beams is verified through finite element method (FEM) under a 100 g acceleration which is loaded on *x*-axis, *y*-axis and *z*-axis, respectively. Moreover, sensor chip is wire-bonded to a printed circuit board (PCB) which contains two identical oscillating circuits. In addition, a steel shell is selected to package the sensor for experiments. Benefiting from the distinctive configuration of the differential structure, the accelerometer characteristics such as temperature drift and cross-interface are improved. The experimental results demonstrate that the cross-interference is lower than 0.03% and the temperature drift is about 18.16 ppm/°C.

## 1. Introduction

Micro-machined accelerometers have been applied in vehicle [1], industrial machinery, and environmental devices due to the merits associated with batch manufacturability, small size, low power and high integration [2,3]. With regard to an accelerometer, the acceleration to be measured is converted into displacement of a movable proof mass, which can be detected using various principles including piezoresistive sensing [4], capacitive sensing [5], resonant sensing [6], etc. Among them, resonant sensing is an attractive method for measuring acceleration [3,7], which has been known for many years based on the resonant principle. The basic principle of resonant sensor is to measure the frequency shift of a resonator, which is affected by the variation of acceleration. Compared with other detection mechanisms, resonant sensing has many advantages such as high accuracy, high sensibility, high resolution, and digital output signal [6,8,9,10].

In recent decades, silicon has been widely used for micro-sensors because of its mature manufacturing process and good material properties. A lot of configurations of silicon resonant sensors have been designed and implemented [9,11,12,13,14,15,16,17] since the first silicon resonant sensor was proposed by Greenwood [18]. Moreover, the conversion of voltage to frequency in surface micro-machined tuning fork oscillators intended as resonant transducers was reported by Roessig T [19]. Generally speaking, the available excitation mechanisms for silicon resonant accelerometers mainly include electrostatic excitation [20], electro-thermal excitation [21] and electro-magnetic excitation [22]. Among them, the electro-magnetic and electrostatic methods require external excitation structures that increase the complexity of the sensor structure and the fabrication process. Thermal stress is induced with regard to the electro-thermal method, which will result in accuracy deterioration. Compared with the silicon material, single crystal quartz is more accordant with the requirement of resonators which can be excited and vibrate in a stable frequency, because of its excellent material properties such as high quality factor, high frequency stability and inherent piezoelectric effect. The first quartz accelerometer was reported by Albert [23]. Since then, it has drawn great attention due to the good performance [24,25,26,27]. Quartz has been widely used as a resonator material in micro-sensors for a long time, and has proven to be more appropriate for the requirements of resonators.

However, most of the conventional quartz resonant accelerometers [25] are composed of metal substrates and quartz resonators. The main drawback is that the metal material with appropriate properties is specially selected and manufactured by the sophisticated precision machining method, which results an increased cost in materials and fabrication methods. Therefore, when designing an accelerometer, the manufacturing cost should be taken into account.

In order to obtain a high-performance, several researches have been done to improve the performance of silicon resonance accelerometer including improving sensitivity, decreasing cross-interference and temperature drift [9,13,28,29]. However, compared with quartz as resonator material, the silicon material properties result in a limitation of performance improvements such as frequency stability, quality factor, and reliability which are quite important for Micro-Electro-Mechanical System (MEMS) inertial sensors applied in the inertial navigation and guidance of aircraft. To solve this problem, a differential accelerometer composed of a silicon substrate and quartz double-ended tuning forks (DETFs) is proposed and fabricated in this work. The proposal has a sound effect on the reduction of cross-interference and temperature drift, which have a significant impact on the performance of resonant accelerometers.

This paper focuses on the design of a new resonant micro-accelerometer composed of a silicon substrate and double quartz DETFs. In order to decrease the influence of cross-interference and temperature drift induced by the thermal stress, a unique differential configuration was designed where paratactic and paralleled quartz resonators are mounted along the diagonal direction of the proof mass. Both the silicon substrate and quartz DETFs were fabricated by MEMS technology, which results in a diminution of the manufacture cost. According to the FEM (finite element method), the length of the flexure hinge which had an effect on the sensitivity of the accelerometer was simulated and analyzed in detail. In accordance with the analysis results, the optimal structure of the resonant accelerometer was obtained. After that, several experiments were carried out to verify the feasibility and reliability of our design. Finally, a discussion was provided to compare with the previous work.

## 2. Sensor Design

### 2.1. Design of the DETF

**Figure 1:**
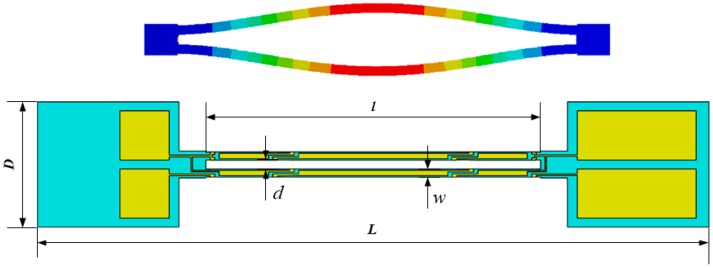
Anti-phase bending model of double-ended tuning fork (DETF).

In consideration of the favorable stress-sensitive property and dynamically balanced structure, a double-ended tuning fork structure is preferred to be used as a resonator [11,30]. The resonator that consists of two identical micro-beams vibrates in an anti-phase in-plane bending model, as shown in Figure 1. When the vibrating beams are regarded as a Euler–Bernoulli beam model and nonlinear terms are ignored, the following equation for the transverse beam oscillation can be obtained:

(1) $$f=f_{0}\left(1+\frac{0.147l^{2}}{Etw^{3}}N\right)$$

where

(2) $$f_{0}=\frac{22.346}{2\pi }\sqrt{\frac{EI}{\rho Al^{4}}}$$

where E is the elasticity modulus of quartz crystal; N is the axial compressive load (applied to the oscillating beam by the proof mass); $f_{0}$ is the unloaded resonant frequency of the DETF; ρ is the density; and w, l, and t is the width, length and thickness of the tines, respectively. Then the differential resonant frequency Δf of the oscillator can be expressed as

(3) $$\Delta f=f_{1}-f_{2}$$

where

(4) $$f_{1}=f_{01}\left(1+\frac{0.147l^{2}}{Etw^{3}}N\right)$$

(5) $$f_{2}=f_{02}\left(1-\frac{0.147l^{2}}{Etw^{3}}N\right)$$

Then, Δf is obtained as

(6) $$\Delta f=(f_{01}-f_{02})+\frac{0.147l^{2}}{Ew^{2}}\left(f_{01}+f_{02}\right)\times \frac{N}{wt}$$

where $f_{01}$ and $f_{02}$ are only related to the structure parameter. Since N is proportional to the external acceleration, one can obtain the value of acceleration by measuring the Δf.

With the bending deflection of the DETF neglected, the relationship between the axial force and axial stress in the beams can be expressed approximately as:

(7) N=wt×σ

where σ is the axial stress in the tines.

Here we assume that the unloaded resonant frequency $f_{01}$ and $f_{02}$ are equal, then substituting Equation (7) into Equation (6), the output of resonant frequency is obtained as:

(8) $$\Delta f=\frac{0.147l^{2}f_{0}}{Ew^{2}}\Delta \sigma$$

where

(9) Δσ=2σ

**Figure 2:**
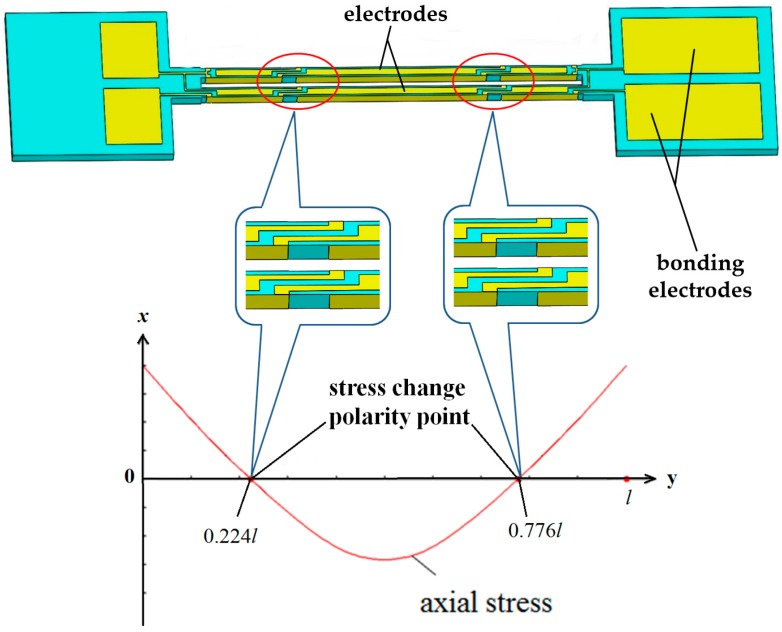
Diagrammatic sketch of the DETF.

Considering the merit of piezoelectric property, *z*-cut single-crystal quartz orientation is adopted in this design, where the width, length and thickness of the tines are parallel to the *x*-axis, *y*-axis and *z*-axis, respectively. To ensure that the resonator vibrates at the pre-established mode, segmented electrodes are deposited on the top, bottom and sidewalls of the beams. Moreover, the boundary of adjacent electrodes is situated in the stress change polarity point [31], as schematically shown in Figure 2.

The DETF resonant frequency is designed by allowing for the manufacture process and the matching oscillation circuit which is usually devised for driving flexural mode vibration below 100 KHz. The detailed values of the dimensions of the DETF are optimized and listed in Table 1. Substituting these parameters into Equation (2), we can get that DETF resonance frequency is 35.309 kHz.

### 2.2. Design of Silicon Substrate

**Figure 3:**
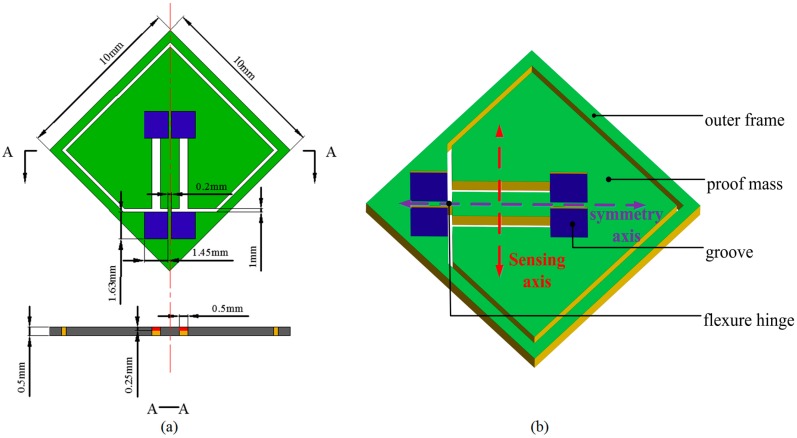
Diagrammatic diagram of silicon substrate: (**a**) front view and cross-sectional view along the A**—**A; (**b**) structure diagram.

As shown in Figure 3, the silicon substrate consists of a proof mass, a flexure hinge and outer frame. In order to mount the DETFs, four grooves are designed, which can guarantee that two DETFs are symmetrical. To reduce the common mode errors in the sensor, such as thermal mismatches, cross-sensitivity and nonlinearity, a differential matched resonator configuration is employed. The unitary proof mass for the resonator can decrease error due to imprecision during manufacturing compared with the ones with separated poof mass. Additionally, the dimensions of the silicon structural parameters are shown in Figure 3a.

**Figure 4:**
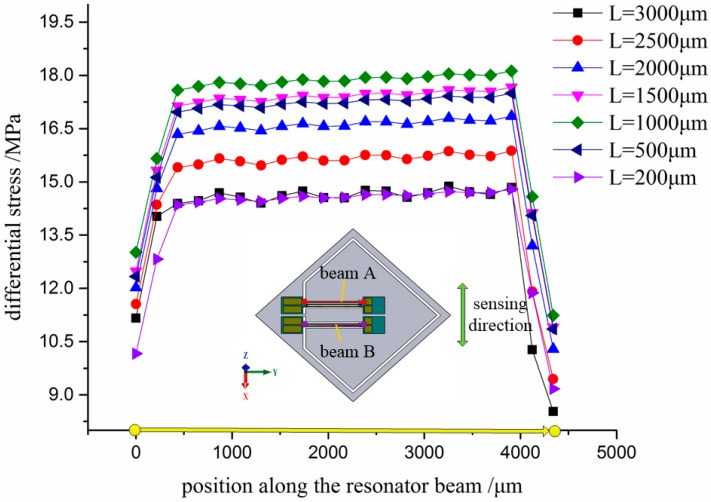
Differential stress distribution along the selected resonator beam versus length of flexure hinge (width is 200 μm; thickness is 500 μm).

**Figure 5:**
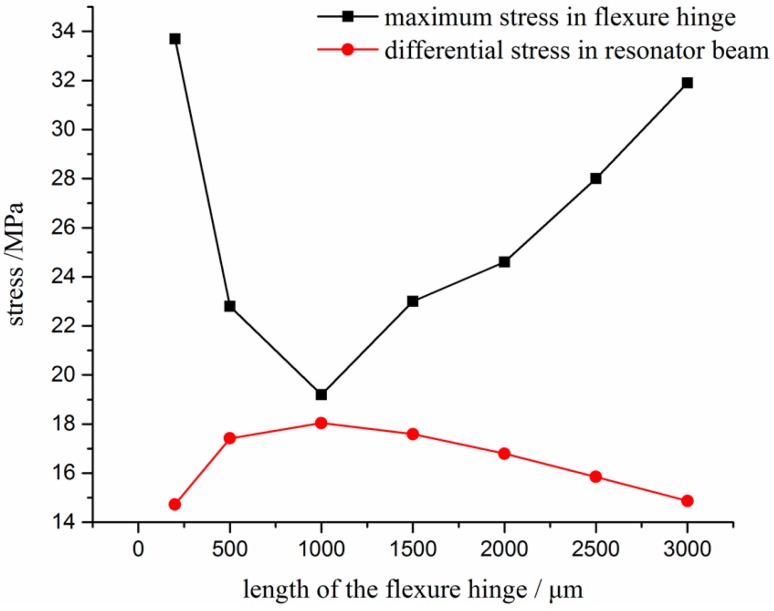
Maximum stress versus different length of flexure hinge (width is 200 μm; thickness is 500 μm).

The most important component of the silicon substrate is the flexure hinge. With a view to the reliability of deep plasma etching process, the width of the flexure hinge is a constant value of 200 μm. Thus, only the length of the flexure hinge was simulated and analyzed through the FEM in detail. The maximum stress in flexure and resonator beam versus length of the flexure hinge was studied as shown in Figure 4 and Figure 5. The results demonstrate that the variation of the maximum differential stress between the double DETFs is in inverse relationship with that of the maximum stress in flexure hinge versus the length of flexure hinge. Then, the optimal length of flexure hinge is 1000 μm.

**Figure 6:**
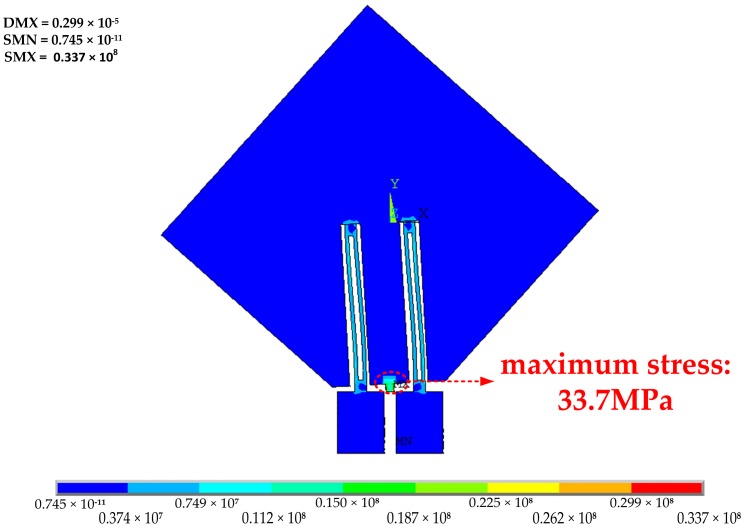
The maximum stress in flexure hinge under 100 g acceleration.

To obtain high sensitivity and ensure the linearity and precision of the accelerometer, the maximum stress should not exceed 1/5–1/6 of the stress limit of silicon [12]. In other words, the stress should be less than 500–600 MPa. As shown in Figure 6, when 100 g acceleration is loaded on the sensing direction, the maximum stress is generated on the flexure hinge with a value of 33.7 MPa, which is far less than 500 MPa. That is to say, the performance of structure is reliable and security.

### 2.3. Operational Principle

**Figure 7:**
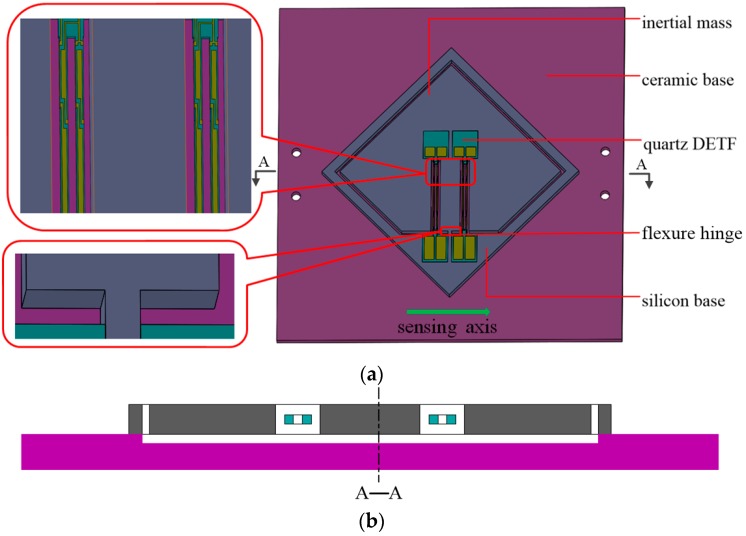
(**a**) Diagrammatic sketch of the resonance sensor; and (**b**) cross-sectional view along A-A.

The resonant uniaxial accelerometer reported in this paper is schematically shown in Figure 7a, which is composed of double DETFs, a ceramic base and a silicon substrate including a movable proof mass. The symmetrical DETFs act as the sensitive element, which are located on both flanks of the flexure hinge and parallel to etch other. It can be found that the paratactic and paralleled quartz resonators mounted along the diagonal direction of the proof mass results in a reduction of the size of sensor.

The outer frame is stabilized by ceramic base and the proof mass is kept free and moveable. To ensure the proof mass is secure and movable when acceleration is applied, a large groove is devised on the front of ceramic base as shown in Figure 7b. The DETFs are driven by external circuit to oscillate at its resonant frequency. The ceramic base with four through-holes serves as a segregation board as shown in Figure 7a. The terminal pins are allowed to pass through the holes and reach the same height with the resonators. Thus, the electrical connection between the bonding pads of DETF and PCBs can be realized by golden wire bonding on the upper end-surfaces of binding posts.

When acceleration is applied along the sensing axis, the proof mass will transfer force to DETFs, which causes an opposite variation in their resonant frequencies. In other words, when one DETF is under tensile stress, the other is under compressive stress. Two DETFs undergoing opposite axial forces can improve the performance of the sensor. The symmetric differential configuration response to spurious effects of thermal loading is similar, which can be reduced by detecting the differential frequency. Additionally, inelastic effect causing pre-stress in the resonators is also decreased.

**Figure 8:**
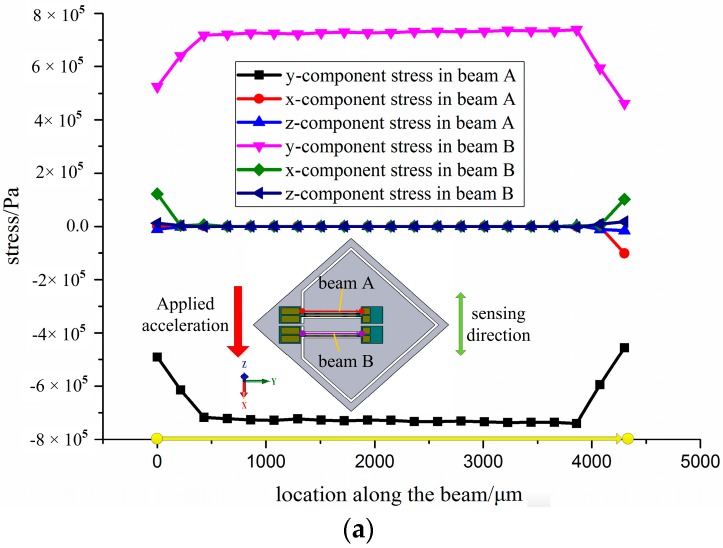

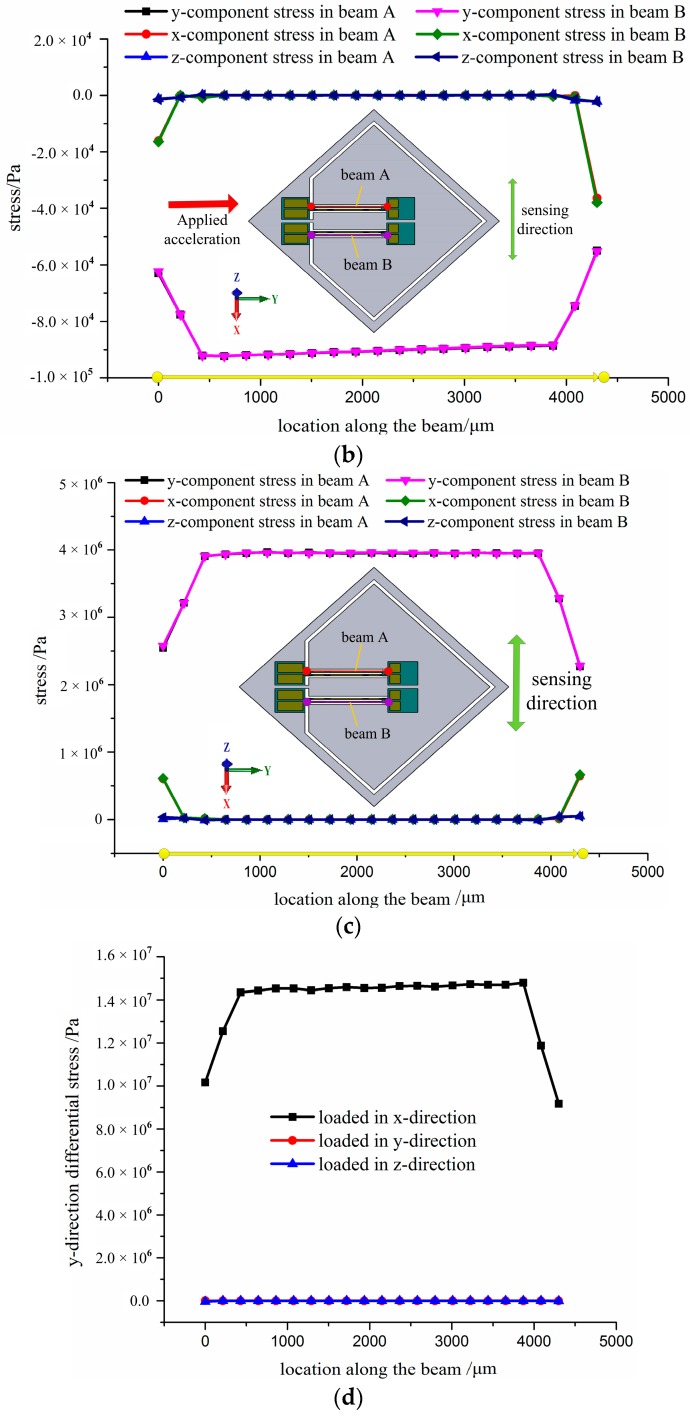
Simulation results of stress in the DETF beam along the selective pass: (**a**) loaded in *x*-direction; (**b**) loaded in y-direction; (**c**) loaded in z-direction; and (**d**) differential stress along the axial direction of the EDTFs.

The anticipative range of the proposed sensor is ±100 g. The stress in the DETF beam along an interior path selected specially is obtained by finite element method, when 100 g acceleration applied to the *x*-axis, *y*-axis and *z*-axis, respectively, as shown in Figure 8 and Table 2. It can be seen that the maximum stress can be obtained when acceleration applied to the sensing directions (*x*-axis). The sensor is insensitive to the acceleration applied to other directions (*y*-axis and *z*-axis). This means that the differential configuration is conductive to the diminution of the cross-interference and improving the performance of the accelerometer. Substituting the stress of FEM simulation in Equation (8), the frequency shifts 1319 Hz for 100 g, and the sensor sensitivity is 13.19 Hz/g.

## 3. Fabrication

### 3.1. Fabrication of Silicon Substrate

**Figure 9:**
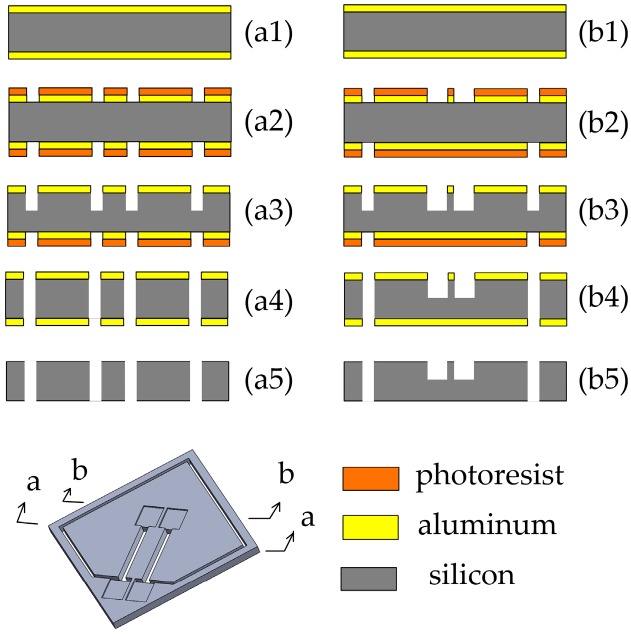
The schematic of the main fabrication process of silicon substrate; (1) Deposit aluminum layers; (2) Photoetching and Etching the aluminum layers; (3) Etching the silicon from the front side; (4) Etching the silicon from the back side; (5) Removing the aluminum layers.

**Figure 10:**
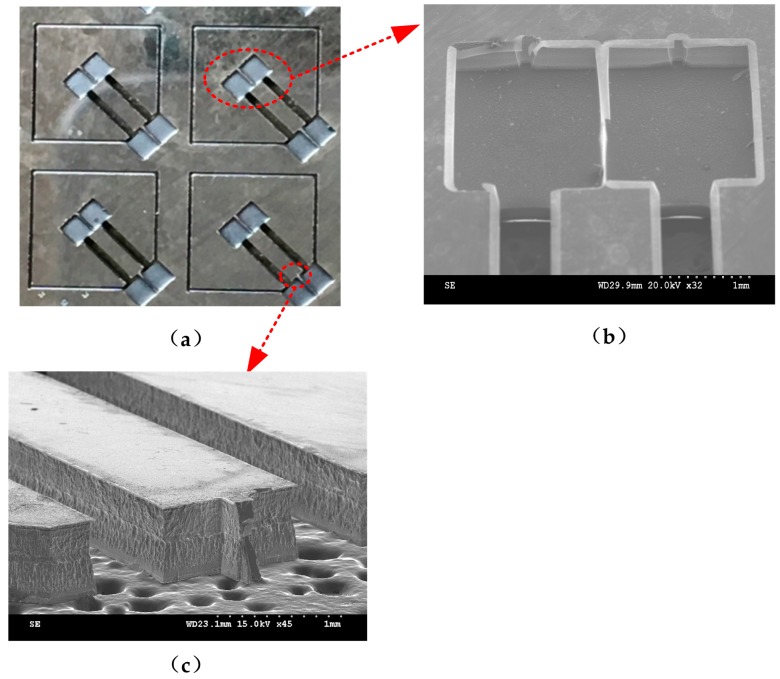
Fabricated silicon substrate and the partial enlarged view: (**a**) fabricated silicon substrate; (**b**) the partial enlarged view of the grooves; and (**c**) cross-section view of the flexure hinge.

The silicon substrate was fabricated by bulk-micromaching technology, and the fabrication process is shown in Figure 9 (a1~a5 are cross-sectional views along a-a, and b1~b5 are cross-sectional views along b-b). First, a (100) oriented n-type silicon wafer whose thickness was 500 μm was selected to deposit aluminum layers with a thickness of 300 nm on the double polished surfaces of the wafer using magnetron sputtering processes. Subsequently, both layers were patterned with a photoresist resist film. After that, the inductively coupled plasma (ICP) was utilized to etch the front side of the wafer with a depth of 300 μm, and then the same process was carried out on the back side. Thus, recess holes and flexure hinge were formed successfully. Finally, the aluminum layers were removed by wet etching. The fabricated silicon substrate is shown in Figure 10.

### 3.2. Fabrication of Quartz DETF

**Figure 11:**
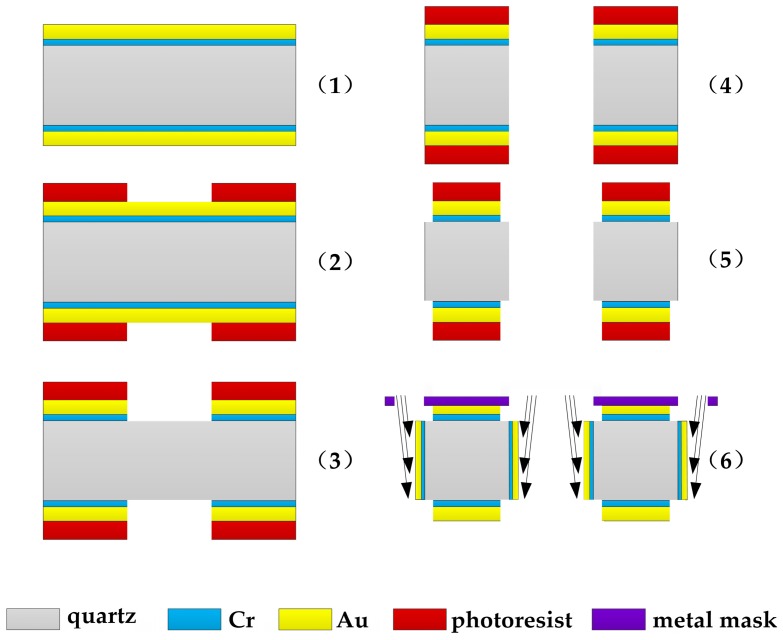
The schematic of the main fabrication process of quartz DETF; (1) Sputtering Cr/Au layer; (2) Photoetching; (3) Etching the Cr/Au layer; (4) Etching the quartz; (5) The fabrication of electrodes on the planar surface; (6) Depositing the sidewall electrodes.

**Figure 12:**
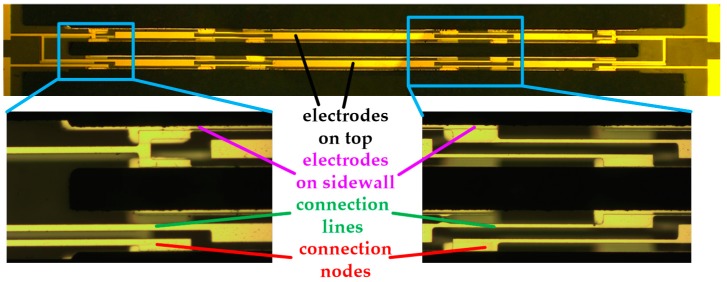
Manufactured quartz DETF and the partial enlarged view.

The quartz DETF was fabricated by wet etching technology due to the fact that it was easy and inexpensive to implement. The main fabrication process is shown in Figure 11. First, a *z*-cut quartz wafer with a thickness of 100 μm was chosen to deposit Cr/Au layer with thicknesses of 50 nm and 200 nm. The Cr layer acted as a transition layer to increase the adhesion of the Au layer. Second, lithography was utilized to pattern the photoresist. After that, the Cr/Au layer was etched by the conventional etchants, respectively. Subsequently, the fluoride-based solution (40%HF: 40% (NH)_4_F = 1:1) was applied to etch the quartz to form the vibration beam. Then, the fabrication of electrodes on the planar surface was implemented using photolithographic technology and Cr/Au wet etching method. Finally, the sidewall electrodes were deposited with a thin metal mask. The manufactured quartz DETF is shown in Figure 12. The connection nodes were designed to connect the electrodes on the planar and sidewall surface.

## 4. Experiment Results and Discussion

### 4.1. Packaging Process

To ensure the reliability and sensitivity, the resonator was assembled on the grooves of the silicon substrate by adhesive bonding. A kind of epoxy-phenolic adhesive (M-Bond 610) with low viscosity (solids content 22%) and available widest temperature range (−269 °C to 230 °C) was specially selected and utilized, which exhibited favorable thermal and mechanical properties and was appropriate for high-performance applications, including high-accuracy transducers. Extremely thin, hard, void-free gluelines (capable of gluelines <0.005 mm) minimized the creep, hysteresis, and linearity problems. Additionally, to guarantee the positional accuracy, four recess holes were formed on silicon substrate which also can be applied to ensure that the double DETFs were symmetry about the flexure hinge. Furthermore, a flip chip bonding machine was employed in the bonding process for alignment and heat treatment. With this equipment, the resonator was adjusted to be aligned with the corresponding recess holes. Afterwards, the chip was heated to 125 °C, followed by the annealing which was employed to eliminate or minimize the residual stress in the structure.

**Figure 13:**
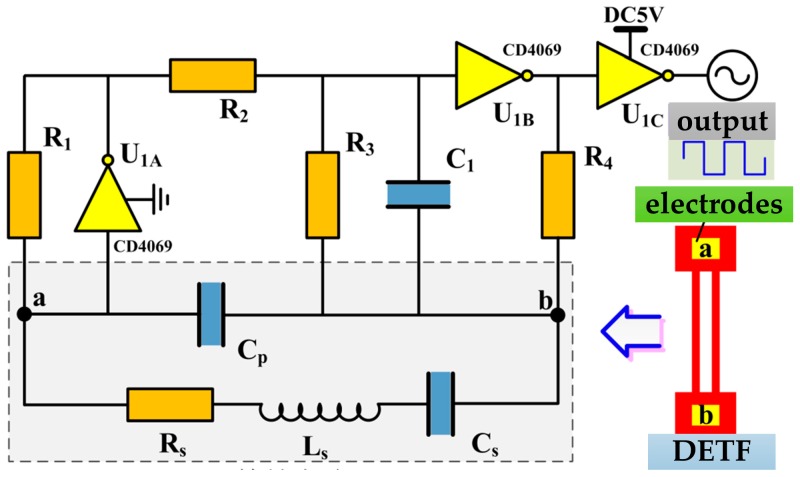
Self-excitation circuit and output square wave.

**Figure 14:**
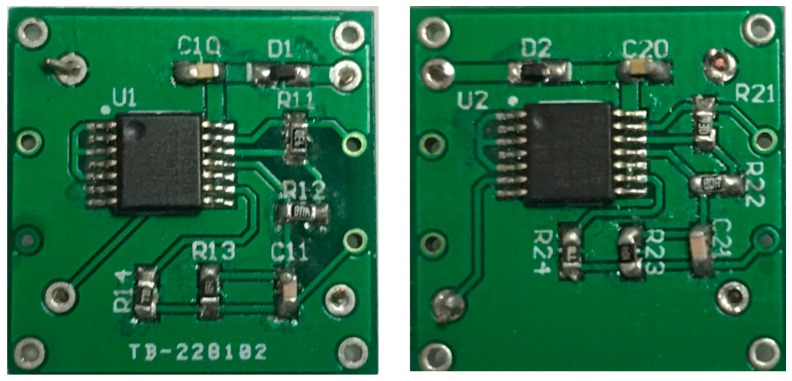
The printed circuit board (PCB) with two oscillating circuits.

By taking the advantages of piezoelectric properties of quartz crystal, the resonant accelerometer was excited through a self-excited circuit which was connected to the electrodes deposited on the DETF’s tines. The self-excited circuit mainly is composed of two phase inverters as shown in Figure 13. Resistors R_1_, R_2_, R_3_ and R_4_ ensure that both the inverters functioned at the linear amplification condition. Capacitance C_1_ was used to eliminate high-frequency harmonics. This circuit offers advantages such as a simple configuration, stable performance, as well as a wide adjustment range for the parameters of electronic components. With the circuit parameters R_1_ = 900 kΩ, R_2_ = 2.56 kΩ, R_3_ = 100 kΩ, R_4_ = 100 kΩ, and C_1_ = 10 pF, the circuit could oscillate stably, and the output square wave was recorded by an oscilloscope. Two same excitation circuits were designed and fabricated on both sides of a printed circuit board (PCB) as shown in Figure 14, which can be used to excite the DETFs into resonant frequency.

**Figure 15:**
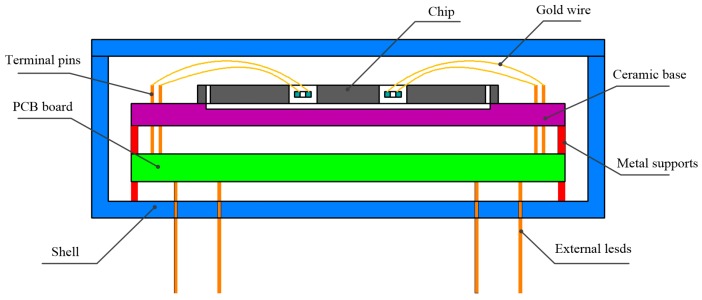
The schematic of the packaging sensor.

**Figure 16:**
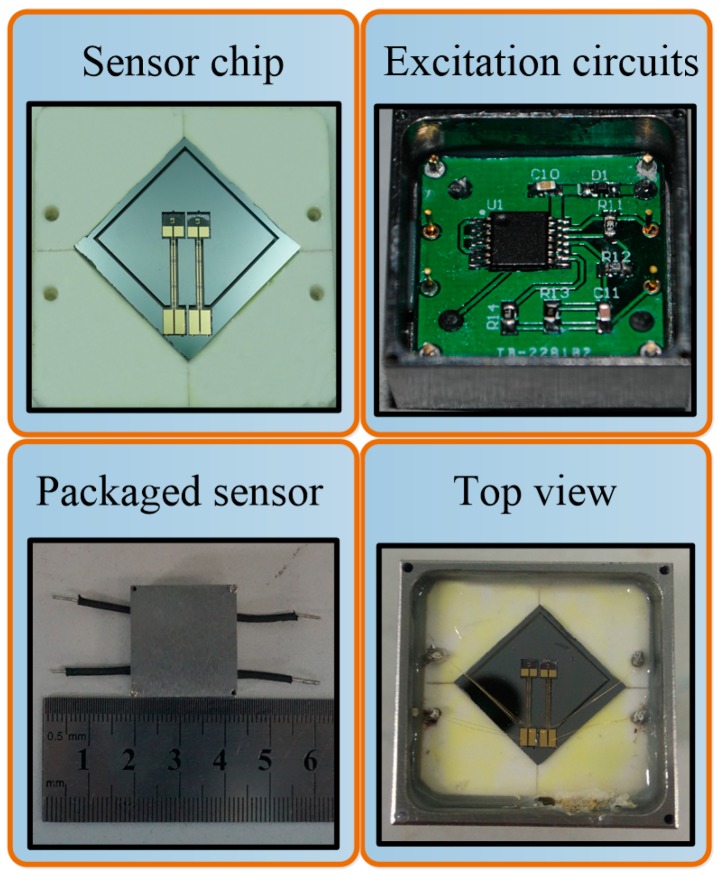
The prototype of packaged sensor.

The sensor chip and PCB were packaged in a stainless steel shell for characterization, as shown in Figure 15. First, the chip needed to be bonded with ceramic base to guarantee the proof mass is movable. Terminal pins were employed to connect the bonding pads on the DETF with excitation circuits through wire bonding technology, and then metal supports were utilized to maintain the chip at horizontal level, which had a crucial influence on sensor performance. The electrical connection between the PCB and test equipment was achieved by external leads. Finally, the chip was sealed in the shell which was filled with standard atmosphere. The prototype of sensor in packaging process is shown in Figure 16.

### 4.2. Static Experiments

#### 4.2.1. Tumbling Experiments

**Figure 17:**
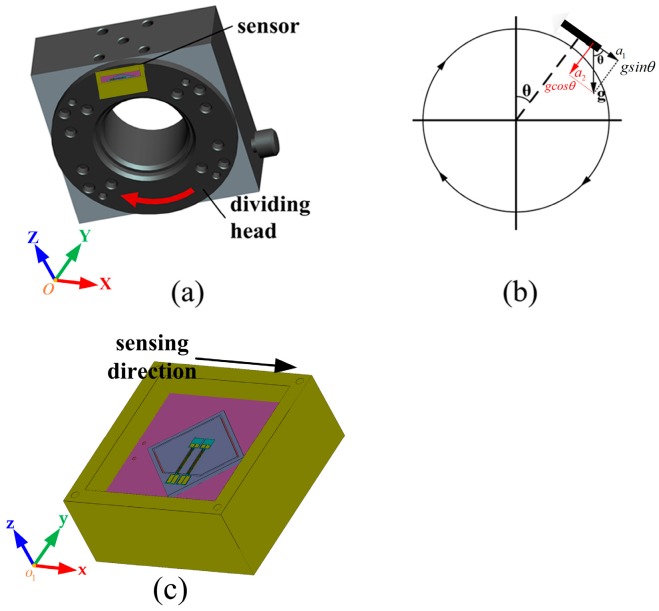
Schematic diagram of tumbling experiment: (**a**) structure of the dividing head and the sensor assembly; (**b**) changing process of acceleration along the sensor’s sensing direction; and (**c**) schematic diagram of the testing sensor.

To evaluate the characteristics of the packaged resonant accelerometer, including sensitivity, nonlinearity, hysteresis, output fluctuation and temperature drift, a precise dividing head with a minimum resolution of 0.01° was employed to load ±1 g acceleration to the sensor, as shown in Figure 17. When the dividing head rotates from 0 to 360° in the earth gravitational field, the acceleration applied on the sensor can be converted into two components: $a_{1}$ and $a_{2}$, which can be represented as

(10) $$\{\begin{array}{c} a_{1}=g\sin\;\theta \\ a_{2}=g\cos\;\theta \end{array}$$

where g is the gravitational acceleration and θ is the rotation angle. Thus, the acceleration loaded on the sensing direction of the accelerometer changes from −1 g to 1 g.

**Figure 18:**
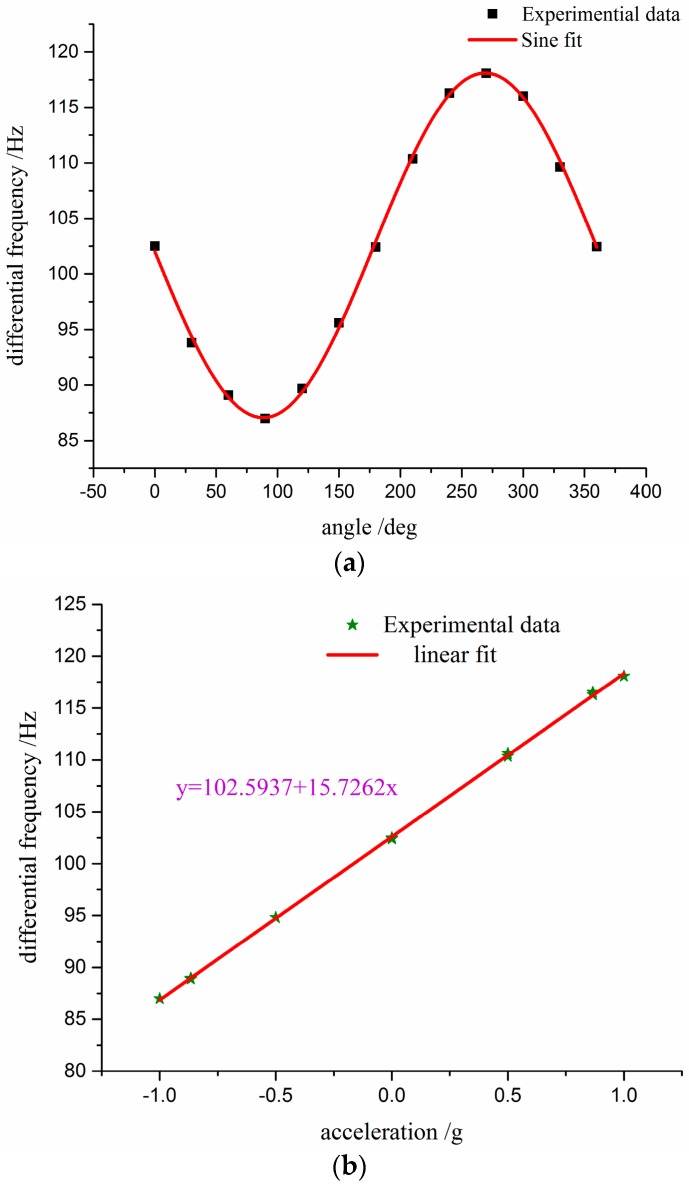
Tumble testing with a precise dividing head: (**a**) differential frequency shift versus rotating angle; and (**b**) differential frequency shift versus applied acceleration.

The static testing results at room temperature are shown in Figure 18. It can be observed that the curve fitted though experiment data is the sine curve in Figure 18a. That is to say, the sensor is insensitive to acceleration in the non-sensitive direction, which corresponds to the simulation results in Section 2.3. Figure 18b shows the original output frequency of the sensor versus the x-axial acceleration. The sensitivity is 15.7262 Hz/g with a maximum nonlinearity of 0.073 %FS, and the initial differential frequency in the absence of applied acceleration is 102.5937 Hz.

**Figure 19:**
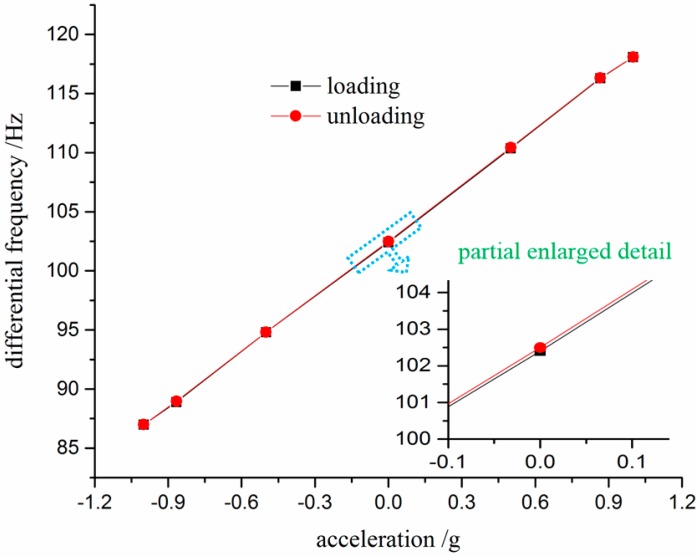
Loading and unloading process.

As shown in Figure 19, the results indicate that the hysteresis is 0.0143 %FS. It can be found that the static characteristics of proposed accelerometer are stable and credible, which are particularly associated with the symmetry of double DETFs. In other words, the design of grooves on silicon substrate is indispensable, which contributes to the improvement of the performance and achieves a high accuracy.

#### 4.2.2. Static Stability Test

**Figure 20:**
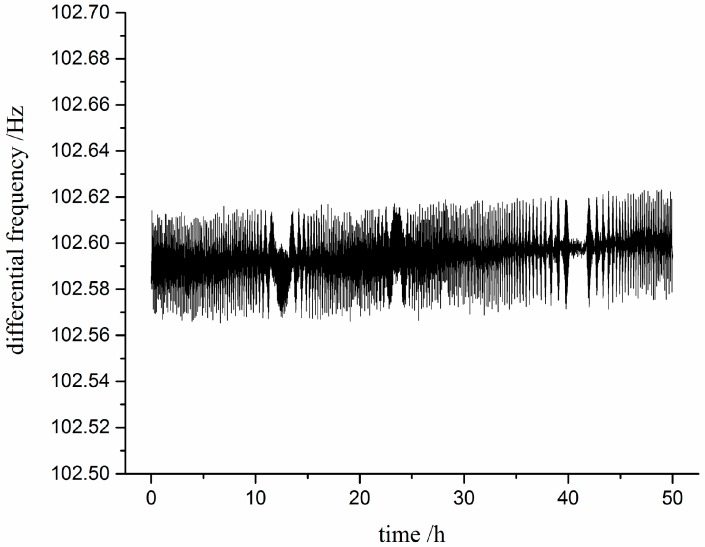
The fluctuation of differential frequency in 50 h.

The static stability test was performed with a thermostat to maintain temperature at 25 °C. As shown in Figure 20, it is clearly seen that the differential frequency variation is about 0.05 Hz in 50 h. The result indicates that the differential configuration is conductive to the improvement of the static stability, compared with the sensor reported by Cun Li [16] (the frequency variation is 0.5 Hz). Additionally, the trend of output frequency shows a quite slow increase which is possibly induced by heat accumulation of the resonator in vibration process.

#### 4.2.3. Cross-Interference Test

**Figure 21:**
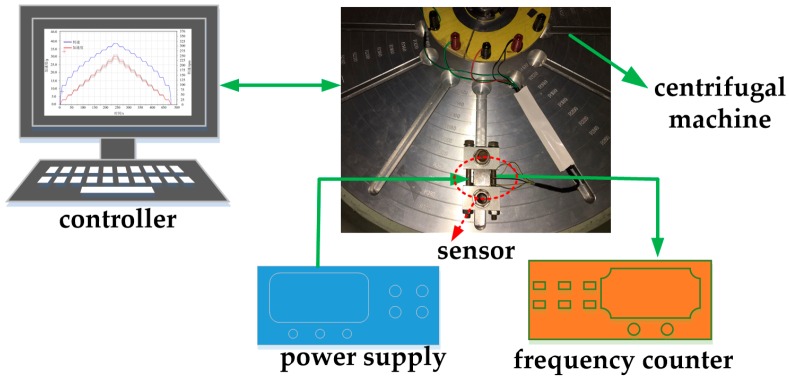
Static measurement system under ±100 g acceleration.

**Figure 22:**
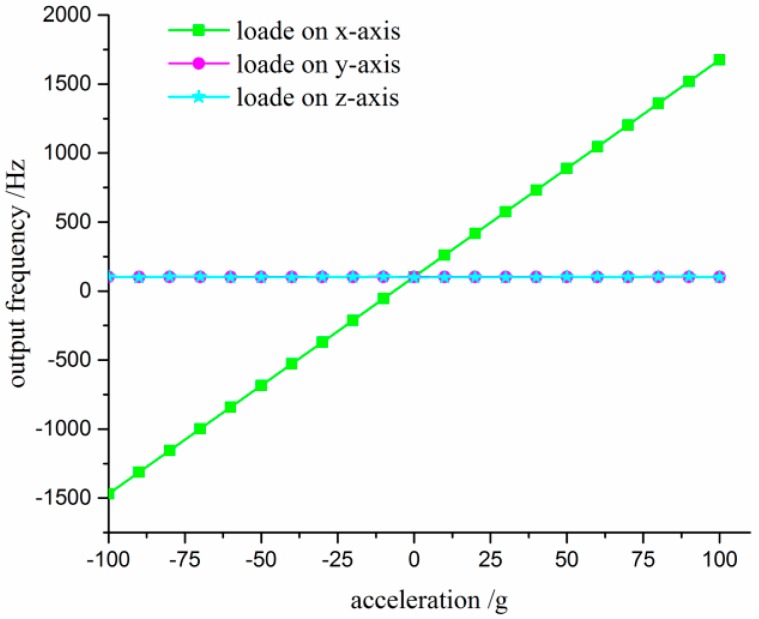
The graph of the original output frequency versus the acceleration loaded in three axes.

An experiment was carried out with a stable acceleration centrifugal machine which was used for conducting the static experiment under ±100 g acceleration as shown in Figure 21. Three cases were implemented with the tested sensor mounted onto the rotary plate with its sensing axis (*x*-axis), *y*-axis and *z*-axis parallel to plate radius, respectively. Acceleration up to 100 g with a 10 g interval was applied to the sensor. Figure 22 shows the sensor’s original output frequency versus the accelerometer loaded on the *x*-axis, and the experimental results are also presented when the sensor is under *y*-axis and *z*-axis accelerations. The sensitivity of the designed device is approximately 15.9 Hz/g, and the initial differential frequency in the absence of applied acceleration is about 102 Hz. As shown in Figure 22, the sensitivity for *y* axis and *z* axis are 0.47 Hz/g and 0.36 Hz/g, which are less than 0.03% and 0.023% of the prime-axis sensitivity. The sensitivity of simulation calculation is 13.19 Hz/g mentioned in Section 2.3. This error may be caused by the fabrication and package process.

#### 4.2.4. Test of Temperature Drift

**Figure 23:**
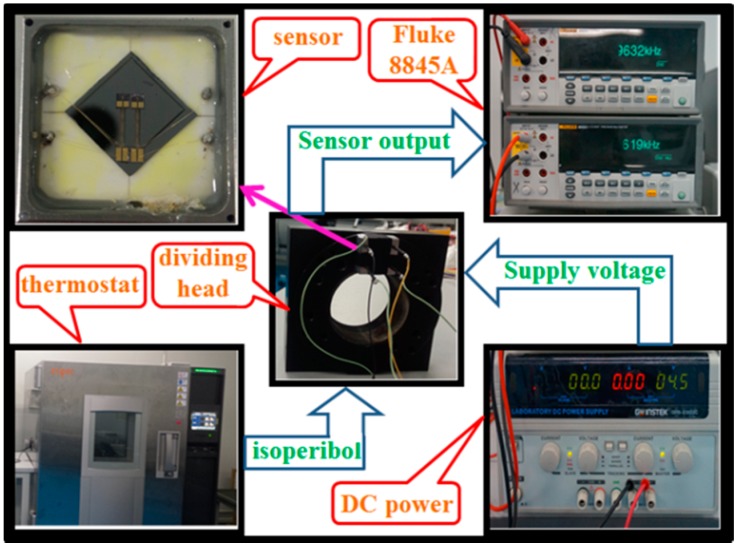
Experimental system of static testing by rotating sensors on a dividing head.

The experimental setup for static test was established as shown in Figure 23. A thermostat (the fluctuation of the temperature is less than 0.1 °C) was utilized to maintain constant temperature in the testing process to verify that the differential structure can reduce the temperature drift. The power supply for excited PCB is 4.5 V and the measured sensor was mounted onto the dividing head with its sensing axis (*x*-axis) parallel to horizontal direction.

**Figure 24:**
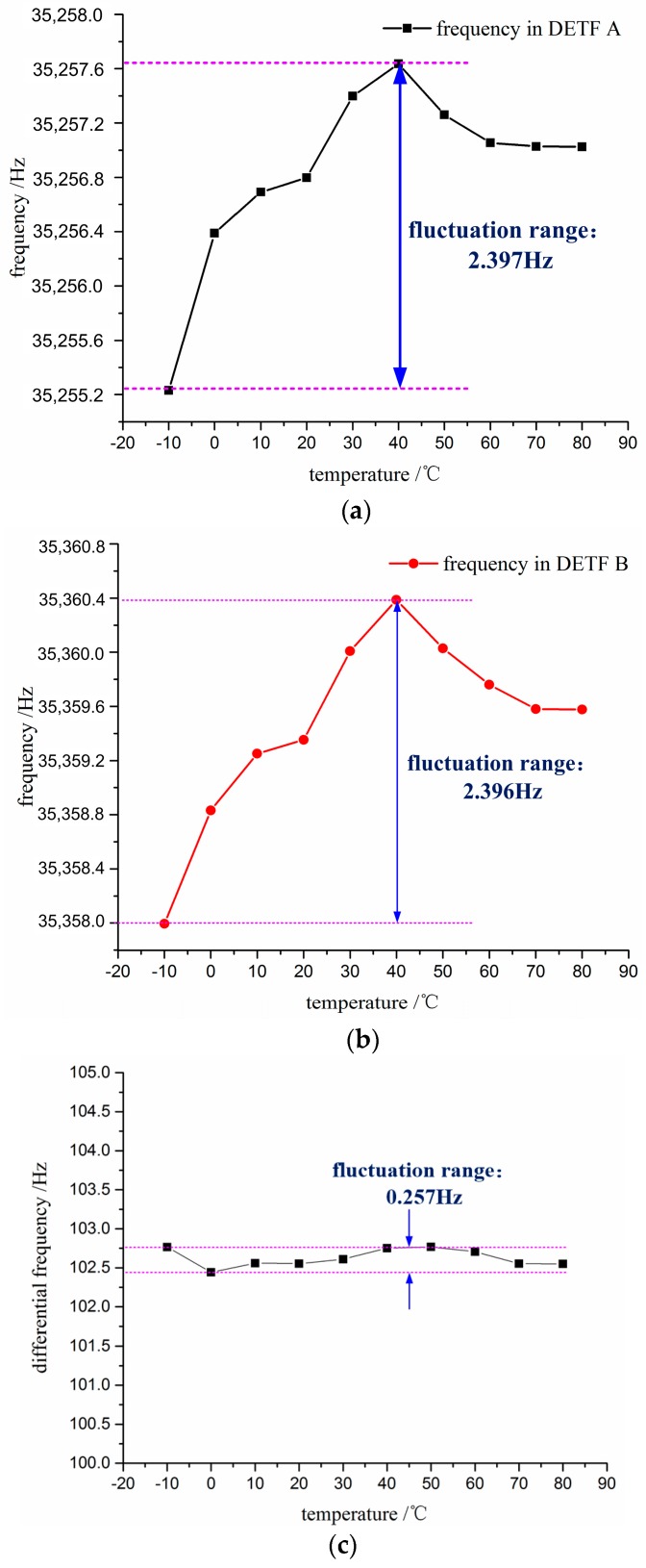
Experimental data of temperature drift: (**a**) temperature drift in DETF A; (**b**) temperature drift in DETF B; and (**c**) temperature drift of the differential frequency.

The temperature drift within a range of temperature from −10 °C to 80 °C is about 0.051 %FS as shown in Figure 24. Compared with the temperature drift of single DETF, the differential temperature drift reduces to 0.257 Hz. As shown in Figure 24a,b, the fluctuation curves are similar. Meanwhile, the temperature corresponding to the peak point is about 40 °C, which is consistent with the characteristic of *z*-cut quartz crystal. The curve shown in Figure 24c indicates that the fluctuation range of differential temperature drift is narrow. The experiment results indicate that the differential structure response to effects of temperature variation can be reduced effectively, which is beneficial to improve the performance of the accelerometer.

### 4.3. Discussion

**Figure 25:**
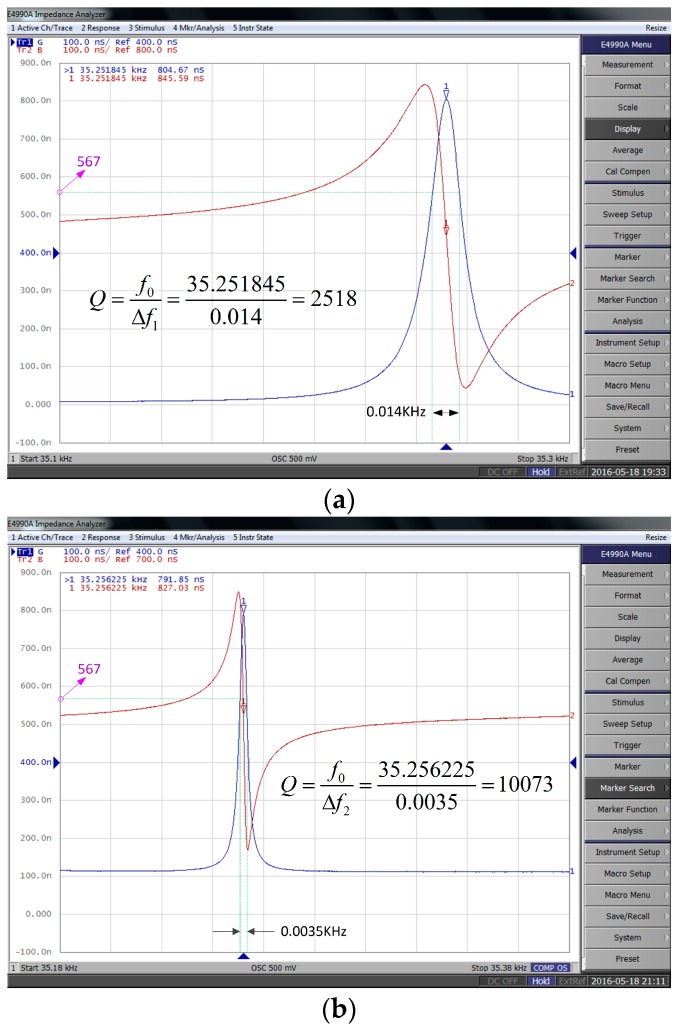
Impedance test results of quartz DETF: (**a**) impedance test results in the air; and (**b**) impedance test results in the low vacuum chamber.

Because of the effect induced by the vapor and fluctuating air on the resonant frequency of DETFs, several researchers have considered the effect of packaging methods on the performance of resonant accelerometers [6,32,33]. Therefore, it is necessary to investigate vacuum packaging effect on the performance of the sensor. The greatest challenge is to achieve vacuum packaging, which is required for MEMS inertial sensors to obtain a high Q value and necessary for navigation grade performance [33]. A low vacuum chamber was employed to avoid the influence of the external environment and provide the pressure about 100 Pa at room temperature (the minimum pressure of the chamber is 100 Pa). The output signal was detected by an impedance Analyzer (E4990A, KEYSIGHT). The results indicate that a higher Q value can be gained under the condition of low vacuum in contrast with that in the air, as shown in Figure 25. Hence, the vacuum package is an effective method to improve the performance of the accelerometer. However, due to the limitation of package equipment utilized in our study, the vacuum package is currently infeasible.

The sensor proposed in our paper is an improved structure design, which leads to a significant reduction in the cross-interference and temperature effect. Table 3 shows the characteristics of the proposed accelerometer compared with the previously published literatures. Compared with other devices, the device proposed in this work can obtain a higher performance such as cross-interference and temperature drift, although the non-linearity is slightly worse than that reported by Cun Li et al. [16] with a single DETF. According to the temperature test result in Section 4.2.4, the performance will deteriorate due to the thermal stress induced by the fluctuation of the temperature. It can be seen that the fluctuation range of the single DETF is about 2.4 Hz which is greater than that of the differential structure (0.257 Hz) introduced in our work. Additionally, the non-differential structure is impossible to eliminate the cross-interference. It can be found that the proposed accelerometer obtains a more favorable comprehensive performance compared to the previous works if considering the non-linearity, cross-interference and temperature drift together. The novel structural design is a promising solution to the accelerometers with favorable comprehensive performance.

## 5. Conclusions

In this work, a differential resonant sensor composed of quartz DETFS and silicon substrate was designed to detective acceleration with low cross-interference and temperature drift. The optimal dimensions of the flexure hinge were determined through the FEM. The differential configuration reported in our paper leads to a significant reduction in the cross-interference and temperature effect. Meanwhile, the experimental results also demonstrate the design. The test results show that the sensitivity of the designed resonant accelerometer is 15.7262 Hz/g with a maximum nonlinearity of 0.073 %FS. The maximum cross-interference and temperature drift are 0.03% and 18.16 ppm/°C. In future work, an investigation of the origin of the drift will be carried out to improve the performance of the sensor. In addition, the data acquisition systems based on the Field Programmable Gate Array (FPGA) will be constructed.

## Figures and Tables

**Table 1:** Dimensions of structural parameters of DETF.

Parameters	Total length *L* (mm)	Total width *D* (mm)	Length of the Tines *l* (mm)	Width of the Tines *w* (μm)	Space between the Tines *d* (μm)	Thickness of the Tines *t* (μm)	Initial Frequency (kHz)
Values	7.5	1.4	3.55	90	100	100	35.309

**Table 2:** The results under 100 g acceleration applied to the *x*-axis, *y*-axis and *z*-axis.

Direction of the Acceleration Loaded	Maximum Differential Stress of *x*-Component (MPa)	Maximum Differential Stress of *y*-Component (MPa)	Maximum Differential Stress of *z*-Component (MPa)
*x*-axis	0.00048	14.7	0.00036
*y*-axis	0.0009	0.00025	0.00065
*z*-axis	0.00014	0.000172	0.000354

**Table 3:** Performance comparison with the existing devices.

Devices	Non-Linearity (%)	Cross-Interference (%)	Temperature Drift (ppm/°C)
Cun Li et al. [16]	0.0019	/	/
Bo Yang et al. [15]	4.49	5.67	/
Hong Ding et al. [13]	/	4.3	/
Comi, C et al. [17]	1	4	29
This work	0.073	0.03	18.16
